# The pancreatic expression database: recent extensions and updates

**DOI:** 10.1093/nar/gkt959

**Published:** 2013-10-25

**Authors:** Abu Z. Dayem Ullah, Rosalind J. Cutts, Millika Ghetia, Emanuela Gadaleta, Stephan A. Hahn, Tatjana Crnogorac-Jurcevic, Nicholas R. Lemoine, Claude Chelala

**Affiliations:** ^1^Centre for Molecular Oncology, Barts Cancer Institute, Queen Mary University of London, Charterhouse Square, London EC1M 6BQ, UK and ^2^Molecular GI-Onkologie (MGO), University of Bochum, Germany

## Abstract

The Pancreatic Expression Database (PED, http://www.pancreasexpression.org) is the only device currently available for mining of pancreatic cancer literature data. It brings together the largest collection of multidimensional pancreatic data from the literature including genomic, proteomic, microRNA, methylomic and transcriptomic profiles. PED allows the user to ask specific questions on the observed levels of deregulation among a broad range of specimen/experimental types including healthy/patient tissue and body fluid specimens, cell lines and murine models as well as related treatments/drugs data. Here we provide an update to PED, which has been previously featured in the Database issue of this journal. Briefly, PED data content has been substantially increased and expanded to cover methylomics studies. We introduced an extensive controlled vocabulary that records specific details on the samples and added data from large-scale meta-analysis studies. The web interface has been improved/redesigned with a quick search option to rapidly extract information about a gene/protein of interest and an upload option allowing users to add their own data to PED. We added a user guide and implemented integrated graphical tools to overlay and visualize retrieved information. Interoperability with biomart-compatible data sets was significantly improved to allow integrative queries with pancreatic cancer data.

## INTRODUCTION

Pancreatic cancer is almost invariably lethal and remains one of the most devastating human malignancies (1). A multitude of studies have been dedicated to elucidating its pathogenesis, resulting in the generation of an increasing volume of data. Hence, there is no shortage of pancreatic cancer raw data but an urgent need for bioinformatics tools to allow robust and rigorous data mining, integration and analysis.

The pancreatic expression database (PED) (2,3) overcomes these challenges and allows the international community to assess and exploit the high volume of pancreatic cancer data to maximal advantage. Here, we provide an update to PED, describing its increased data content and several new structural, design and visualization enhancements from PED 2.0 reported in 2011 (3) to the current PED 3.0 release. These updates further expand the utility of PED for pancreatic cancer research.

## NEW DEVELOPMENTS

### Improved data structure, content and connectivity

**Figure 1.**
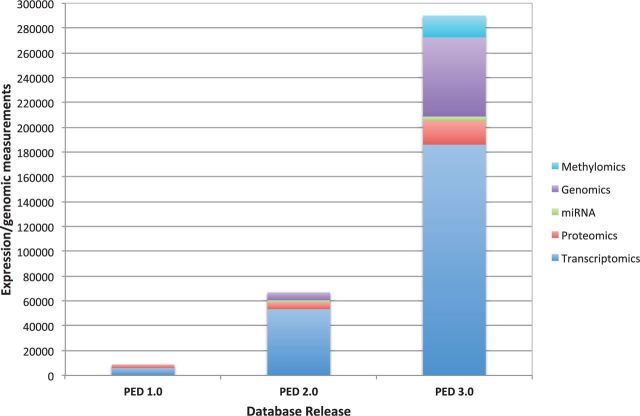
Data growth in PED. Data for the three releases are shown.

Many new data sets were added to PED (Figure 1). Currently, PED 3.0 holds 153 data sets comprising >250 000 expression/genomic measurements, which is four times larger than the previous release. PED 3.0 database structure and existing content underwent major revisions. We have introduced an extensive controlled vocabulary and expanded the data content to cover differential methylomics alongside transcriptomic, proteomic, microRNA (miRNA) and genomic studies. We have expanded PED connectivity with external databases. We also have added data from large-scale meta-analysis studies and included an option allowing users to add their own data to PED. A detailed comparison between features of PED 2.0 and 3.0 is presented in Table 1.

**Table 1.** Summary of PED features

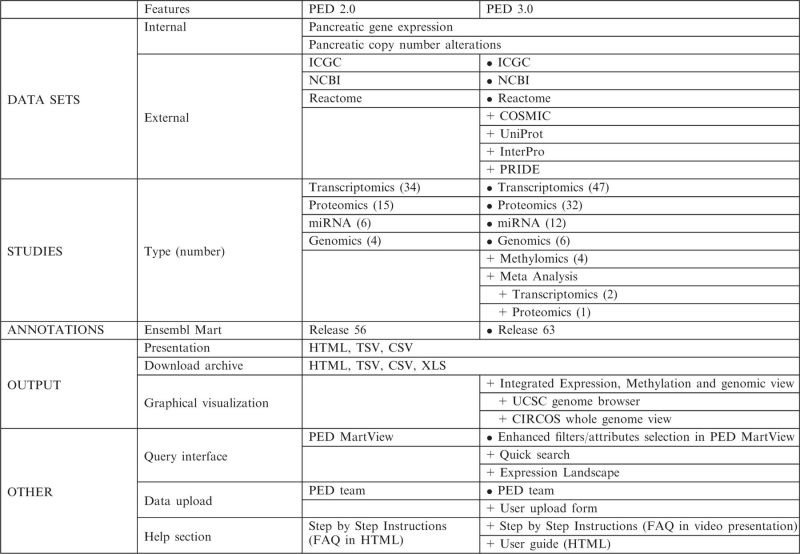

• Updates to existing features

+ New features in current release

#### Data structure

Similar to the previous release, PED data are divided into two separate data sets. The ‘Gene Expression Data Set’ contains relevant data from transcriptomic, proteomic, methylomic and miRNA studies, and the ‘Copy Number Alteration Data Set’ contains copy number alteration (CNA) data from genomic studies.

The underlying structures of these data sets have changed significantly to accommodate detailed information on the experimental design and presentation. Each data set is now conceptually divided into three separate parts:

- *STUDY*: contains basic information about the published articles.
- *EXPERIMENT*: contains detailed information about the experimental design. It uses an extensive controlled vocabulary to record specific details of the samples used in each study including experiment focus, platform technology, types of target and baseline samples, computational pipeline, etc. One study may correspond to multiple experiments.
- *EXPRESSION (CNA)*: contains lists of genes/proteins/probesets/miRNAs (genomic regions) reported to be expressed/regulated (altered) in the specific experiments. Depending on the availability of data in the published article, we also store additional information such as fold-change, *P*-value, expression site (copy-number, log2ratio, frequency) and validation method.

Data from the original articles are manually curated, reviewed for accuracy and consistency and loaded into PED. The gene expression data set is built around the Ensembl human gene annotations (4), which enables direct integration of heterogeneous pancreatic-related data with a rich collection of public annotations such as genes, proteins, Single Nucleotide Polymorphism (SNP) information, sequences, gene structures and multi-species data. To avoid annotation errors, all data are mapped at the transcript level using the pre-established Ensembl annotations and microarray probe set mapping (Ensembl release 63). Where possible, obsolete or discontinued probe identifiers are mapped to current identifiers. All CNA data are presented on the GRCh37/hg19 genome version. Genomic data conversions between assemblies are done, where necessary, using the LiftOver tool from the UCSC Genome Browser (5).

#### Data content

In the latest version, PED 3.0 represents pancreatic-centred data extracted from 99 published studies representing 153 experimental data sets. The database contains 226 135 differential or expression measurements and 63 894 DNA CNAs on a large number of specimen types, including pancreatic tissues and body fluids collected from healthy subjects and patients with benign, precancerous and malignant disease states, pancreatic cancer cell lines and xenograft models under different treatment conditions. This is over four times larger than the previous version (Figure 1).

In addition to the transcriptomic, proteomic, miRNA and genomic data, the data collection pipeline has been enhanced to cover differential methylomic and large-scale meta-analysis studies. Altogether, the PED 3.0 data collection now describes pancreatic disease expression measurements and related-regulation events in 11 992 genes/proteins, 56 878 transcripts and 428 miRNAs as well as methylation events in 2440 genes and 15 072 transcripts. The CNA section includes information on 32 039 gains, 23 991 losses, 1053 deletions, 4719 amplifications and 125 Loss of Heterozygosity events occurring in distinct genes and genomic areas.

#### Data upload

Until now, our team selected the data sets for inclusion in PED. In responding to a long-standing request from our users’ community, we have added an upload function for researchers to upload their own published data sets to be included in PED. To encourage participation, the submission process is kept simple; no registration nor login requirement is imposed, although users need to provide a valid email address so that we can contact them in case there are issues/questions regarding the uploaded data set. Our team will check the submitted data and perform necessary modifications or additions required for the inclusion of the uploaded data into PED.

#### Data connectivity and interoperability

In addition to NCBI (6), we also provide direct access to a number of BioMart-compatible data sets available through BioMart interface (7,8) such as ICGC (9,10), Reactome (11), COSMIC (12), UniProt (13), PRIDE (14) and InterPro (15) to allow integrative queries with PED data sets. This integration allows researchers to quickly extract related information about pancreatic cancer genes and genomic regions such as the pathways they control, overlapping somatic mutations, functional analysis of the resulting proteins or a broad range of proteomics data.

### Improved query and data retrieval

#### Filter and attribute selection

The basic PED request-response architecture through our customized version of MartView remains the same. However, with our enriched data content, the interface has been greatly improved with an enhanced structure for filters and attributes to accommodate elaborate yet precise queries. This greatly improves and facilitates data navigation and retrieval across the experimental data sets. Independent experimental validation of microarray experiments is presented, where available.

The specimen/sample information has been significantly improved and refined. An extensive controlled vocabulary is used to record fine-grained details of the samples used in each experiment. This provides users with more control over the selection of experimental data types. For two-group (differential expression) studies, specimen/sample information is segregated into a target group and a baseline/control group to better reflect the experimental designs. This offers a better representation and facilitates the query of cancer specimens versus normal control, or specimens that are treated versus non-treated. A comprehensive list of changes in filters and attributes presentation from PED 2.0 to this current release 3.0 is provided as Supplementary Material (Supplementary Table S1, Supplementary Table S2).

#### Quick search

The home page offers the option to quickly extract summarized information about a gene/protein of interest using HGNC/Ensembl gene, miRNA accession or SwissProt/Ensembl protein identifiers. The search box has an autocomplete function. As users type, it will try to complete the search term by suggesting possible matching terms. The results contain a summary of the studies reporting a link between the search term and pancreatic cancer. The retrieved information includes study type, experimental questions, platform technology, target and baseline samples used, observations/findings, corresponding fold-change and *P*-value as well as validation method (if any). This allows users to quickly validate or check the current knowledge about the role of a gene/protein in pancreatic cancer.

#### Graphical visualization

**Figure 2.**
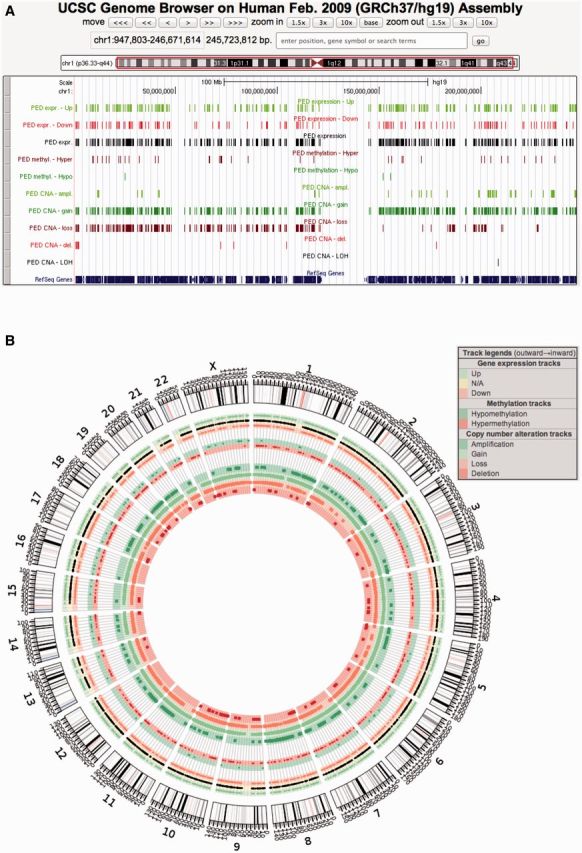
Integrated graphical view of transcriptomic and genomic changes observed between pancreatic ductal adenocarcinoma and normal tissue samples. This query starts by choosing the ‘Pancreatic Expression Database’ from the ‘Choose Database’ from the drop-down selection in the right panel. A ‘Choose Dataset’ drop-down selection menu will automatically appear. In this example, users will choose ‘Pancreatic Gene Expression’ data set. The next step involves choosing the appropriate filters to restrict the query in this first data set by clicking on the ‘Filters’ node on the left, expanding the filter section ‘EXPERIMENT:’, selecting ‘PDAC study—tissue’ and ‘Pancreatic ductal adenocarcinoma (PDAC)/Normal pancreas (NP)’. The overlapping CNAs are added as a second data set by clicking on the second ‘Dataset’ from the bottom of the left panel and choosing ‘Pancreatic Copy Number Alteration’ from the ‘Choose additional dataset’ drop down menu that will appear in the right panel. Clicking the ‘Results’ button on the left side of the toolbar will retrieve a result table. To generate the graphical view of the results, simply hit the UCSC or CIRCOS buttons on the right side of the toolbar. (**A**) Single chromosome (chr 1) view in UCSC Genome Browser. (**B**) Whole-genome view in CIRCOS viewer.

We have implemented integrated graphical tools to PED MartView to allow users to query, overlay and visualize retrieved information using UCSC Genome Browser (5) and CIRCOS viewer (16). Based on the selection criteria, a separate browser window displays differentially expressed genes and/or CNA regions in the UCSC genome browser under different tracks (Figure 2A). Users can choose to change the chromosomal view by selecting a chromosome from the drop-down list provided. A simple color-coding scheme is used to present up/down-regulated genes and different types of CNAs. Users can also opt for a whole genome view generated by the CIRCOS software (Figure 2B). To provide additional flexibility, users can click on a particular chromosomal band in the static image to be redirected to the UCSC Genome Browser for a detailed view of the region of interest.

### Improved website and documentation

We have completely redesigned the web interface and improved its functionality by using modern web technologies. The website home page now contains a quick search option for users to quickly investigate the relationship between their gene/protein of interest and pancreatic cancer. In addition to the updated filter and attribute grouping, the PED Mart query interface has been reconfigured with additional visualization options. Another query interface has been created for interested users to view the published results from the largest meta-analysis on the pancreatic cancer expression landscape (17). An extensive user guide has been added to describe all the features and functionalities of the website. Additionally, FAQs have been added with short video presentations. A glossary section has been included in the user guide to describe the filter and attribute groups specific to PED. For users who may not be familiar with the abbreviations used for different experimental features, an explanatory list of acronyms is also provided.

## FUTURE DIRECTIONS

The amount of pancreatic cancer data is growing continuously. Though PED provides a good coverage of the cancer-related changes in the human model, there is a considerable amount of experimentally derived information available in the pancreatic cancer mouse model as well as in single gene studies that need to be integrated and made easily accessible. New findings are also being discovered from next-generation sequencing. Thus, implementing new modules to handle these new data types will be our next target. We also plan to further develop the analytical aspect of PED to allow comparative analysis of alterations in human tissues, cell lines and mouse models as well as survival analysis and basic data processing. An ultimate goal is to integrate and present all levels of details from single-gene studies and therapeutic information.

## SUPPLEMENTARY DATA

Supplementary Data are available at NAR Online.

## FUNDING

Cancer Research UK [programme grant reference 15310]; Funded by breast Cancer Campaign (to R.J.C.). Funding for open access charge: Cancer Research UK [15310].

*Conflict of interest statement*. None declared.
